# Bifidobacterial genes upregulated by resistant starch investigated using multi-omics have orthologs in infant gut isolates

**DOI:** 10.1093/ismeco/ycag136

**Published:** 2026-05-17

**Authors:** Molly E Millar, Miriam Abele, Hannah C Harris, Todor T Koev, Andrea Telatin, Raymond Kiu, Douwe Van Sinderen, Yaroslav Z Khimyak, Christina Ludwig, Lindsay J Hall, Frederick J Warren

**Affiliations:** Food, Microbiome and Health, Quadram Institute Bioscience, Norwich Research Park, Norwich, NR4 7UQ, United Kingdom; Wellcome Sanger Institute, Hinxton, CB10 1RQ, United Kingdom; Bavarian Centre for Biomolecular Mass Spectrometry (BayBioMS), TUM School of Life Sciences, Technical University of Munich, Freising, 85354, Germany; Food, Microbiome and Health, Quadram Institute Bioscience, Norwich Research Park, Norwich, NR4 7UQ, United Kingdom; School of Chemistry, Pharmacy and Pharmacology, University of East Anglia, Norwich Research Park, NR4 7TJ, United Kingdom; Food, Microbiome and Health, Quadram Institute Bioscience, Norwich Research Park, Norwich, NR4 7UQ, United Kingdom; Centre for Microbial Interactions, Norwich Research Park, Norwich NR4 7UG, United Kingdom; Food, Microbiome and Health, Quadram Institute Bioscience, Norwich Research Park, Norwich, NR4 7UQ, United Kingdom; Department of Microbes, Infection and Microbiomes, School of Infection, Inflammation and Immunology, College of Medicine and Health, University of Birmingham, Birmingham, B15 2TT, United Kingdom; Institute of Microbiology and Infection, University of Birmingham, Birmingham, B15 2TT, United Kingdom; APC Microbiome Ireland & School of Microbiology, University College Cork, Cork, T12 YN60, Ireland; School of Chemistry, Pharmacy and Pharmacology, University of East Anglia, Norwich Research Park, NR4 7TJ, United Kingdom; Bavarian Centre for Biomolecular Mass Spectrometry (BayBioMS), TUM School of Life Sciences, Technical University of Munich, Freising, 85354, Germany; Food, Microbiome and Health, Quadram Institute Bioscience, Norwich Research Park, Norwich, NR4 7UQ, United Kingdom; Department of Microbes, Infection and Microbiomes, School of Infection, Inflammation and Immunology, College of Medicine and Health, University of Birmingham, Birmingham, B15 2TT, United Kingdom; Institute of Microbiology and Infection, University of Birmingham, Birmingham, B15 2TT, United Kingdom; Norwich Medical School, University of East Anglia, Norwich, NR4 7TJ, United Kingdom; Food, Microbiome and Health, Quadram Institute Bioscience, Norwich Research Park, Norwich, NR4 7UQ, United Kingdom

**Keywords:** Bifidobacteria, resistant starch, microbiome, CAZymes

## Abstract

*Bifidobacterium* species and strains are key members of the human gut microbiota, appearing soon after birth and persisting into adulthood. Resistant starch is an important dietary substrate for adult-associated bifidobacteria, where its fermentation supports host health. However, less is known about how different starch structures interact with bifidobacteria. Here we show that growth kinetics and gene expression differ depending on starch structure. Using detailed growth assays, genomics, and metabolomic analyses, bifidobacterial starch hydrolysis capabilities were closely associated with their CAZyme profiles. In one isolate of *Bifidobacterium globosum*, we identified a gene cluster encoding three multi-functional amylase enzymes complemented by several starch-binding modules, the genes and proteins of which were significantly upregulated in response to starch. Homologs of genes in the cluster were found in the genomes of bifidobacterial isolates from weaning infants providing insights into their role in the maturation process of the microbiota. Uncovering mechanisms of metabolic interaction between starch structures and bifidobacteria underscores the importance of this ecological function and potential health implications.

## Introduction

The taxonomic and functional changes in the human gut microbiota during early life have long-term impacts on health [1]. Gut microbiome assembly begins shortly after birth, and is influenced by a variety of factors [2]. Bifidobacteria, as early colonizers of the infant gut, play a key role in health [3–5]. For breastfed infants, human milk oligosaccharides (HMOs), which cannot be digested by the host, are selectively fermented by certain bifidobacteria, contributing to microbiome development [2, 6, 7]. The weaning period, when solid foods are introduced, presents a critical stage for microbiome maturation [8]. This transition often involves starchy foods [9] containing resistant starch (RS)—starch which is incompletely digested and reaches the colon [10]. Since the intestinal microbiome of infants is still developing, starch fermentation plays a role in gut microbiome ecological dynamics and maturation [9]. Fibre fermentation produces beneficial metabolites, such as short-chain fatty acids (SCFAs) including acetate and butyrate [11], contributing to health [12, 13]. Bifidobacteria possess a unique carbohydrate utilization pathway termed ‘bifid shunt’, leading to the release of primary metabolic products such as lactate and acetate [14]. Bifidobacteria can be administered in specific settings as probiotics for infants, particularly in preventing gastrointestinal conditions such as necrotizing enterocolitis [15].

As important members of the developing gut microbiome, several *Bifidobacterium* species interact with RS [16]. Bifidobacteria can act as primary degraders of starch and facilitate cross-feeding with other gut microbes, influencing overall gut ecology [17–19]. An *in vitro* study using infant faecal inocula demonstrated an increased relative abundance of *Bifidobacterium* post-weaning in the presence of a high amylose maize starch (HAM) [20]. During weaning, *Bifidobacterium* must utilize at least one of the following: HMOs; solid food-derived glycans like starch, and/or host-derived mucins. *Bifidobacterium longum* is particularly key to the transitional weaning period [21–23].

Starch granules consist of amylose and amylopectin, and properties such as crystalline structures [24] and amylose content [25] affect starch structure. Maize starches vary in composition from normal (20%) to high (70%) amylose which causes structural differences due to the propensity of amylose to recrystallize into a gel more readily after cooking to form RS3 [26]. RS degradation requires multiple specialized enzymes and carbohydrate-binding modules (CBMs) that target crystalline supramolecular structures [16, 19, 27, 28]. Gene profiles of mammalian microbiomes are frequently enriched with relevant carbohydrate-active enzymes (CAZymes) which are specific to host carbohydrate intake and ecological niche—demonstrating a close host-bacteria co-evolutionary relationship [29]. The first gene identified in bifidobacterial starch degraders called *apuB* encodes a bifunctional amylopullulanase [30]. With evidence of heated and cooled starch (a process which promotes RS development) degradation in *Bifidobacterium adolescentis* [31], Jung *et al.* have since reported several multi-domain resistant-starch degrading enzymes in *B. adolescentis* and *Bifidobacterium choerinum* [32], as well as performed growth studies on a bovine *Bifidobacterium pseudolongum* isolate [33]. An additional ecological study of the *B. pseudolongum* starch utilization phenotype was carried out showing that the species may play a critical role in efficient microbiome-starch interactions [34]. Though, few studies encompass bifidobacteria from different niches grown in different types of RSs.

To address these knowledge gaps, we investigated RS utilization in 11 bifidobacterial belonging to 7 species identifying enzyme profiles, mechanisms of utilization, and functional conservation in the bifidobacterial genus, including species and strains from various hosts and origins. We measured growth kinetics of strains on different starches including metabolomics on a subset of six strains. Combined with genome annotation of hydrolytic enzymes and CBMs, we discovered a cluster of three genes in *Bifidobacterium globosum* which we further validated using transcriptomics and proteomics. We performed transcriptomics and proteomics to interrogate gene regulation in response to RS uncovering a novel gene cluster involved in RS degradation.

## Materials and methods

### Bacterial strains

Strain accessions are detailed in Supplementary Table S1. Unique bifidobacteria (LH strains) were isolated as described previously [3]. Six bacterial strains were provided by the Hall Lab, which were acquired as unique isolates from infant stool from the Baby-Associated MicroBiota of the Intestine study [3]. Note that *Bifidobacterium pseudocatenulatum* LH659-662 strains isolated at Day 159 after birth originate from the same stool sample, with average nucleotide identity (ANI) values between strains >0.9999. Reference strains were purchased from NCIMB (Aberdeen, UK), UCC (Cork, Ireland), and ATCC (Manassas, USA).

### Starch growth assays

All strains were grown at 37°C in either RCM, MRS media, a prepared minimal MRS (mMRS) lacking a carbon source as a negative control, or mMRS prepared with specified carbohydrates. All media were supplemented with cysteine-HCl (0.05% w/v). For starch media, starches were boiled at 100°C in mMRS in a microwave, mixing at regular intervals, for 1–3 min before being sterilized by autoclaving at 126°C for 15 min. Autoclaved starches were cooled to room temperature. To generate bacterial growth curves, 10 μl samples were removed for serial dilution at various time points, plated on MRS agar, incubated anaerobically in a Ruskinn Concept Plus for 2–3 days, and colonies were counted. The data shown are mean values from three biological replicates. Full details of culture conditions are provided in the supplementary materials.

### Bacterial metabolomics

Culture medium aliquots of 600 μl were stored in 1.5 ml snap-cap plastic microtubes at −20°C (if not immediately processed). The aliquots were thawed at room temperature or 4°C overnight. Each sample metabolome was quantified using a Bruker Avance III NMR spectrometer; details are in the supplementary materials. Spectra were analysed using NMR Suite Processor v8.41 (Chenomx®).

### Proteomic data acquisition and data analysis

*B. pseudolongum* subsp. *globosum* NCIMB 702245 (hereafter called *B. globosum* 45) was cultivated for 24 h as described in 10 ml MRS and mMRS media. mMRS was supplemented with carbohydrates: 0.5% D-glucose (Sigma-Aldrich); cooked and cooled starch substrates prepared as described in mMRS with 1% normal maize starch (Sigma-Aldrich). Cultures were centrifuged for 10 min at 4000× *g*. The cells were washed with 5 ml PBS before centrifuging again as before. The cells were resuspended in 100 μl 100% trifluoroacetic acid and incubated in a thermocycler for 5 min at 55°C, which was neutralized with a volume of 900 μl 2 M Tris solution. The lysed samples were quantified by Bradford assay and stored at −80°C. Samples were processed for mass spectrometry according to the SPEED protocol as described previously [35, 36], and described in the supplementary materials.

### Alpha-amylase protein structure prediction

The full-length structure of multi-modular protein discovered in *B. globosum* 45 containing alpha-amylase and CBM74 modules (designated as BpAmy74) was predicted using AlphaFold2 [37] and described in the supplementary materials.

### RNA extraction and sequencing

RNA extractions of *B. globosum* 45 were performed by culturing in 40 ml mMRS supplemented with: 0.5% D-glucose (Sigma-Aldrich), cooked and cooled starch substrates 1% w/v HylonVII®, and 1% w/v normal maize starch. The RNA samples were prepared using RNeasy® Mini kits (Qiagen) to perform the RNA extraction using the manufacturer’s protocol with additional chemical lysis and bead-beating steps detailed in the supplementary materials. RNA sequencing was performed by Azenta, Cambridge, UK, also detailed in supplementary materials.

### Transcriptome data processing

The raw reads were processed as described in the supplementary materials into a raw counts matrix. Using iDep.96, data were visualized and DESeq2 was used to generate differential expression values [38, 39].

### Statistical analyses

Statistical analysis of bacterial growth data was carried out using area under the curve calculations and repeated measures two-way Analysis of Variance (ANOVA) comparing starch to the negative control at each time point, performed in GraphPad Prism v9 (GraphPad Software). Protein-Groups.txt result file from MaxQuant was used as an input for Omicsviewer 1.1.5 [40] to perform t-tests. All other statistical analyses were performed in GraphPad Prism v9.

### Whole bacterial genome sequencing

For whole genome sequencing (WGS) of a reference type strain purchased, *B. pseudolongum subsp. pseudolongum* DSM 20099/NCIMB 702244, the strain was cultured (see supplementary materials) and DNA extracted using FastDNA™ SPIN Kit for Soil, following manufacturer instructions, with extended 3 min bead-beating. DNA sequencing was completed using Oxford Nanopore MinION (see supplementary materials). A draft genome of a single contig containing 1 916 804 bp was generated as described in the supplementary materials.

### Carbohydrate active enzyme and binding module genome annotation

Bacterial isolate WGS were generated for *B. pseudolongum* subsp. *pseudolongum* NCIMB 702244 (hereafter called *B. pseudolongum* 44) or obtained from NCBI [41] (the full details are available in Supplementary Table S1). For unique isolates investigated bioinformatically in this study, three infants (V1, V2, V3) donated at a similar age enabling the isolation of 19 unique isolates [3] (see Supplementary Table S2). The WGS of *B. pseudolongum* 44 and *B. globosum* 45 were scored for ANI using pyani v0.2.7 [42]. dbCAN2 was used for the prediction of glycan substrates for CAZymes, associated CBMs, and gene clusters (CGCs) [43]. Gene alignments were completed using Clustal Omega using default settings to create the alignment and visualized using Jalview (v2.11.4.0); the residues were highlighted manually using Inkscape (v1.4.2).

## Results

### *Bifidobacterium* CAZyme profiles

**Figure 1:**
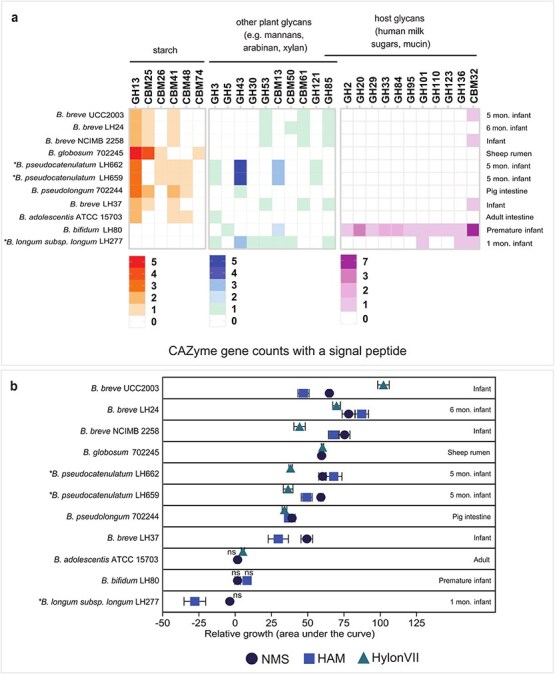
CAZyme profiles and utilization of starches by *Bifidobacterium* isolates from different sources. (a) The number of each CAZyme families and carbohydrate binding modules detected in the whole genomes of each strain was assessed and categorized by substrate (starch, other plant glycans, host glycans). To focus on enzymes involved in extracellular starch hydrolysis, we filtered the results to display only CAZymes with a signal peptide as determined by SignalP. Asterisks denote that isolates were derived from stool donated from the same infant. *Bifidobacterium pseudolongum* subsp*. globosum* NCIMB 702245 referred to as *Bifidobacterium globosum* 702 245 and *B. pseudolongum* subsp. *pseudolongum* NCIMB 702244 and referred to as *B. pseudolongum* 702 244. (b) The growth of bacteria in the presence of starch was compared to growth in the absence of a substrate, where the mean difference (mean diff.) in the AUCs was statistically compared to assess starch utilization in mMRS medium supplemented with 1% w/v: NMS (30% amylose), HAM (50% amylose), or HylonVII® (70% amylose) starch. Not all strains were tested on HylonVII® starch and HAM. The growth of each strain was determined by generating by log10 conversion of the Colony Forming Units (CFU) counts from 3 to 5 time points between 0 and 48 h and calculating the AUC (experiments were carried out in triplicate). The AUC and SEM were calculated and used to perform a Dunnett’s multiple comparisons test between the starch condition and the no substrate control. Error bars denote Standard Error of the Mean (SEM). A positive integer value for mean difference between AUC relative to the negative control indicates growth on the starch substrate; a negative integer indicates no growth. Values are statistically significant unless indicated (ns >0.01). AUC, area under the curve; HAM, high amylose maize starch.

We used isolates collected during a longitudinal infant microbiome study whose ages spanned the weaning period [3, 15] as well as reference strains from established culture collections to represent known starch degraders from other niches (Fig. 1). To focus on extracellular enzymes, we filtered the results to display CAZymes with a signal peptide as determined by SignalP (Fig. 1a). Glycoside hydrolase (GH) families with enzymes involved in starch degradation include GH 13, 14, 15, 31, 57, 119, and 126 [44], of which the assessed isolates only encoded GH13 family enzymes equipped with a signal peptide (full CAZyme profiles can be found in Supplementary Table S3). Additionally, several starch-specific CBMs were identified including CBM 25, 26, 41, 48, and 74. Strains lacking genes encoding a signal peptide-containing GH13 enzyme, as well as relevant starch-related CBMs, were isolated from very young (1 month old) or preterm infants (*Bifidobacterium bifidum* and *B. longum* subsp. *longum*). Instead, these isolates possessed sequences related to host glycan metabolism, particularly HMO degradation (Fig. 1a). Amongst the remaining nine bacterial isolates, *Bifidobacterium pseudocatenulatum* from a weaning-age infant (5–6 months), and two animal isolates were predicted to encode the highest number of GH13 enzymes (Fig. 1a). *Bifidobacterium pseudocatenulatum* isolates were shown to encode several enzymes involved in the degradation of other plant glycans, but no extracellular enzymes related to host glycan metabolism. This suggests there is a need to understand how phenotypes of infant bacteria vary over the weaning period.

### Starch degradation by *Bifidobacterium* isolates from different origins

*Bifidobacterium* strains were tested using multiple starch structures prepared by autoclaving and cooling maize starches containing different amylose contents (20%–70% amylose). *Bifidobacterium pseudocatenulatum*, *B. pseudolongum*, and *Bifidobacterium breve* strains were all able to hydrolyse multiple types of starch (Fig. 1b), consistent with their CAZyme profiles. In contrast, 2 strains—*B. bifidum* LH80, and *B. longum* subsp*. longum* LH277—showed no significant growth on starch (Fig. 1b). We confirm that *B. adolescentis* ATCC 15703 strain had no significant growth on starch (Fig. 1b) [45]. Amongst the various starch types tested, growth values varied, with HylonVII® (70% amylose maize starch) being the least accessible for all strains except *B. breve* UCC2003. *Bifidobacterium globosum* 45, *B. pseudolongum* 44, and *B. breve* LH24 achieved significant overall growth on all types of starch tested (Fig. 1b). *Bifidobacterium pseudolongum* 44, *B. globosum* 45, and the *B. pseudocatenulatum* LH strains showed a higher prevalence of predicted GH13 extracellular enzymes (Fig. 1a). *Bifidobacterium globosum* 45 also encoded a gene with the RS-binding module CBM74 [46], meaning it can be used as a model for further analysis of its growth on these different starch types.

### Starch structure impacts the growth and metabolite output of *Bifidobacterium* in a strain-dependent manner

The impact of starch structure on growth of *Bifidobacterium* isolates was analysed in combination with metabolomics. Molecules generally detected over time included maltose, acetate, ethanol, and lactate. Maltose and acetate were the most consistently detected across strains (Supplementary Fig. S1). Maltose was the primary product of starch digestion released whilst acetate was the main SCFA metabolite indicative of starch metabolism (Supplementary Fig. S1). Maltose and acetate were only compared within-strain as indicators of hydrolysis and metabolism of starch, respectively.

**Figure 2:**
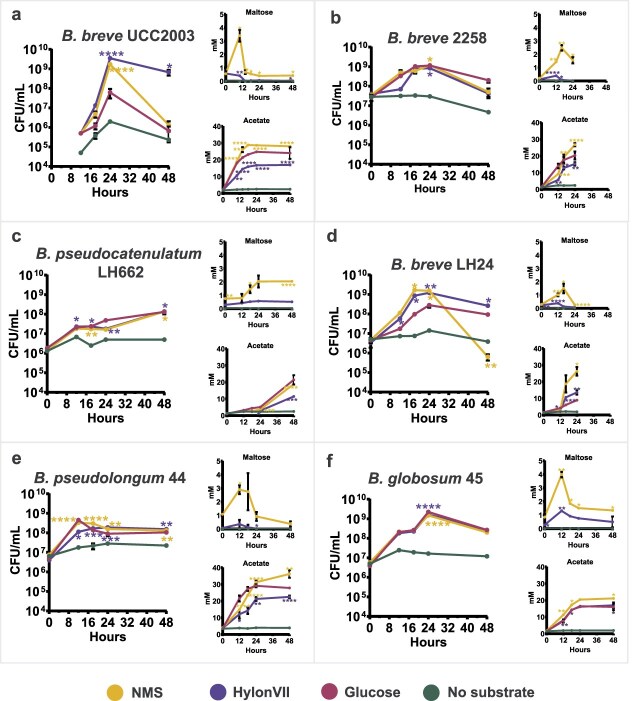
Variation in growth kinetics and metabolite production by Bifidobacterium strains (a-f). Acetate and maltose concentrations were quantified in mMRS with no substrate (negative control), glucose, NMS and 70% amylose maize (HylonVII®) starch. Note that the metabolite concentrations are not biomass-adjusted. Asterisks represent a significant difference (using repeated measures ANOVA) between substrate at each individual time point where ^****^P < .0001, ^***^P < .001, ^**^P < .01, ^*^P < .05. Statistical models were used to ascertain if starch had a significant effect on the growth of each strain relative to the negative control. The same statistical methods were used to analyse the impact of each starch on production of maltose and acetate. Values displayed for each time point are the mean of three biological replicates. Error bars denote SD. Due to unforeseen circumstances the 0 h data point for UCC2003 CFU/mL was lost.

In the presence of normal maize starch (NMS) and HylonVII®, *B. breve* UCC2003 exhibited significantly increased growth, ~4-log higher than the negative control (*P* < .0001) (Fig. 2a) (statistics available in Supplementary Table S4). By 48 h, NMS cell counts were no longer significantly different to the control, whereas cells grown in HylonVII® entered the stationary phase, with counts still significantly higher than the control (*P* = .0115). The difference between cell counts between NMS and HylonVII® was significant (*P* = .0117) at 48 h suggesting that starch structure and amylose content significantly affected growth patterns. When coupled with growth data, an increase in maltose concentration provided evidence of extra-cellular amylolytic activity; we interpreted uptake of maltose when maltose concentration decreased. Maltose concentrations during HylonVII® fermentation were significantly lower over multiple time points than during NMS fermentation (*P* = .0175), which could be due to the more resistant structure leading to slower maltose release. The acetate levels in NMS were significantly higher by an order of magnitude compared to the negative control (*P* < .0001) and the glucose control (*P* = .0007), peaking at 18 h. In contrast, acetate concentrations were significantly lower in the HylonVII® condition compared to NMS at every time point after 18 h (*P* < .0001). Minor replication of *B. breve* UCC2003 in the no substrate condition observed here was not uncommon for reference (lab-adapted) strains; we hypothesize they may have continued replication from their pre-culture trajectory before entering a stationary/lag phase when in the no substrate medium. Similar growth, acetate, and maltose utilization patterns were observed in *B. breve* NCFB 2258 (Fig. 2b), suggesting consistent starch utilization phenotypes in this species.

In contrast, two unique isolates of *B. pseudocatenulatum* LH662 and *B. breve* LH24 derived from the stool of infants aged 5–6 months, exhibited different starch utilization profiles. *Bifidobacterium pseudocatenulatum* LH662 showed significantly increased growth on each type of starch compared to the negative control, yet elicited a lower growth increase compared to *B. breve* LH24 (Fig. 2c and d). *Bifidobacterium pseudocatenulatum* LH662 was in the lag phase for the first 24 h followed by a gradual increase in growth. Maltose concentration in the supernatant increased after 12 h and remained elevated over 48 h, suggesting starch hydrolysis without complete maltose uptake; a phenomenon noted in other starch-degrading bacteria which participate in cross-feeding interactions [47]. In contrast, *B. breve* LH24 was more efficient in degrading NMS and HylonVII® compared to *B. pseudocatenulatum* LH662, increasing its cell count by ~3-log within 24 h. For *B. breve* LH24 in the NMS condition, maltose peaked and then rapidly declined by 24 h, leading to a higher acetate production in the NMS condition.

For animal isolates *B. pseudolongum* 44 and *B. globosum* 45 we observed different utilization phenotypes (Fig. 2e and f). *Bifidobacterium pseudolongum* 44 exhibited less growth on more RS HylonVII® compared to NMS (*P* < .0001 at 12 h (peak growth) time point), and this was reflected in acetate production in the HylonVII® condition (*P* = .0128 at the endpoint of 48 h). In contrast, *B. globosum* 45 displayed the same pattern of growth on both starch types, with no significant difference in cell counts at 24 h (*P* = .7562 at 24 h (peak growth) time point), nor acetate concentrations (*P* = .1201). *Bifidobacterium pseudolongum* 44 achieved its peak growth at 12 h compared to 24 h for *B. globosum* 45. Importantly, *B. globosum* 45 exhibited two exponential growth phases, separated by a short lag phase, during which there was a significant increase in maltose from both NMS and HylonVII®. Compared to *B. pseudolongum* 44 where only NMS caused a significant increase in maltose, we conclude that *B. globosum* 45 is more capable of degrading more RS structures. This unique growth and metabolite production pattern suggested that *B. globosum* 45 was able to more completely hydrolyse HylonVII® starch. Lower end-point acetate concentrations in *B. globosum* 45 (36 mM vs 21 mM) suggest that, despite enhanced hydrolytic capacities, its efficiency in converting sugars into SCFAs was lower.

WGS analysis of *B. pseudolongum* 44 and *B. globosum* 45 indicated an ANI score of 93.4%, indicating that the strains are not members of the same species (nor subspecies), but are closely related [48]. This close relationship, combined with their phenotypic variation, presented an opportunity for further investigation into how they metabolize starch.

### Exploring genomic drivers of *Bifidobacterium*-starch interactions

**Figure 3:**
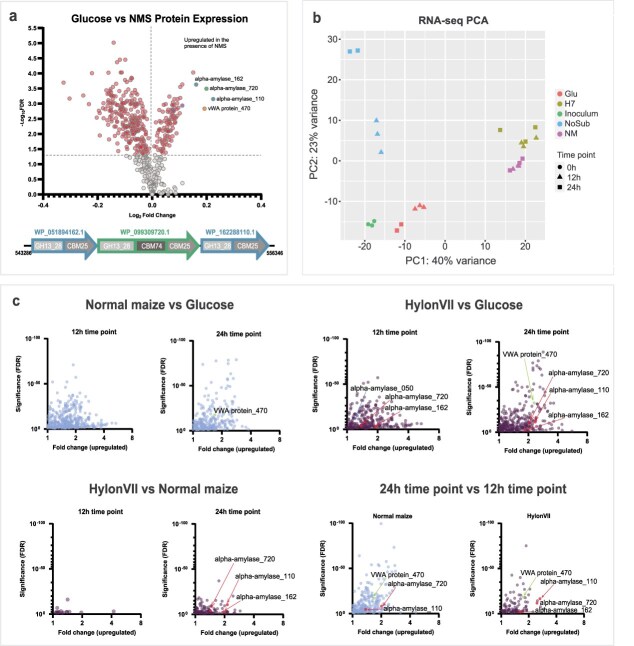
Proteome and transcriptome analysis of *Bifidobacterium globosum* 45. (a) Differential protein production was analysed via *t-*test with multiple-testing correction FDR to measure differentially abundant proteins in the presence of 1% w/v normal maize starch vs 1% w/v glucose after 24 h culture. Bacterial cultures were carried out in triplicate. Significant results are colourized red, significance cut-off defined as FDR < 0.05; − Log_10_(0.05) = 1.301. Proteins of interest are colour-coded corresponding to the following genes: WP_051894162.1 (blue) = alpha-amylase_162; WP_099309720.1 = alpha-amylase_720 (green); WP_162288110.1 = alpha-amylase_110 (blue). The genes are colour-coded with the displayed gene cluster present beneath in the *B. globosum* 45 genome containing 3 Ig-like fold containing proteins which possess α-amylase (GH13) and starch-binding modules. Further proteins of interest are colourized: two pilin-related proteins (purple) and vWA protein_470 (orange). (b) Principal component analysis of RNA expression between substrates of *B. globosum* 45. Plot displays variance of the transcriptome of strains under different conditions and time points in triplicate for the starch conditions and in duplicate for glucose and no substrate conditions. Substrates tested were in the presence of glucose (Glu), normal maize (NM) starch, high amylose HylonVII® maize (H7) starch, and no substrate (NoSub). Time points displayed demonstrate when RNA was extracted: 0 h (inoculum culture of the experiment), 12, and 24 h. (c) Differential genes expression volcano plots display the upregulation of specific genes, using DESeq2 to quantify fold changes in expression and significance value FDR. Genes significantly upregulated were displayed comparing the following culture conditions: normal maize vs glucose, HylonVII® vs glucose, HylonVII® vs normal maize, and 24 vs 12 h time point. Specific genes significantly upregulated such as vWA-domain containing gene and three clustered α -amylase genes are highlighted by colour (if absent, they were not significantly upregulated under that condition).

We exploited the phenotypic differences between *B. pseudolongum* 44 and *B. globosum* 45 to identify genes involved and apply any new information to the unique LH infant isolates also tested. Enzyme annotation using dbCAN revealed that *B. globosum* 45 had a higher number of GH13 family enzyme hits and starch-specific CBMs compared to *B. pseudolongum* 44 (Supplementary Table S5, Fig. 1a). Notable, *B. globosum* 45 possessed various multi-modular proteins with multiple CBMs associated with a single GH13 enzyme, many of which were predicted to be extracellular (Supplementary Table S6). Among the identified proteins, *B. globosum* 45 contained five predicted extracellular α-amylases, compared to three for *B. pseudolongum* 44 (Fig. 1a, Supplementary Table S6). The α-amylases in *B. globosum* 45 included auxiliary starch binding modules such as CBM25, CBM26, and CBM74 (WP_099309720.1, here referred to as alpha-amylase_720), which are absent in *B. pseudolongum* 44. Interestingly, three of these multi-modular proteins were located on the same genomic locus (Fig. 3a), and are not currently designated as α-amylases, but as ‘Ig-like domain-containing proteins’ in the NCBI database (Supplementary Table S7). These three proteins (alpha-amylase_162, alpha-amylase_720, and alpha-amylase_110) are clustered at the same locus, alpha-amylase_720 being the largest (Fig. 3a). We note that these are likely orthologs of similar proteins discovered by Jung *et al.* in *B. adolescentis* [32], though they did not find genes located at the same genomic locus.

### Proteomic analysis of a bifidobacterial isolate with an enhanced RS degrading phenotype

To assess the role of these proteins, we performed proteomic analysis on *B. globosum* 45 cultured in the presence of NMS or glucose. Of 1570 predicted proteins in the genome, 1081 proteins were detected by LC-ESI-MS/MS, and 729 differentially expressed proteins between the two conditions were detected. A total of 619 proteins were significantly upregulated in glucose condition, whilst 110 were significantly upregulated in NMS (Fig. 3a). The most upregulated abundant proteins in the starch condition detected were the three proteins belonging to the gene cluster of Ig-like fold-containing proteins predicted to have α-amylase activity and starch-binding modules: alpha-amylase_162, alpha-amylase_720, and alpha-amylase_110 (Fig. 3a). Whilst these proteins were the most upregulated proteins after exposure to starch, these proteins were 12%–17% upregulated in the NMS condition (with high significance: 1.12-fold change for WP_051894162; 1.15-fold change for WP_099309720; 1.17-fold change for WP_162288110). A vWA-domain containing protein WP_026643470.1 was also upregulated; commonly associated with multi-protein complexes [49] (Fig. 3a). Alpha-amylase_162 and alpha-amylase_720 were shown to be expressed in the glucose condition, but their expression levels were significantly upregulated in the presence of starch (Supplementary Fig. S2). In contrast, alpha-amylase_110 and vWA protein_470, were expressed at much lower levels in the starch condition and were barely detectable in the glucose condition (Supplementary Fig. S2).

Modest increases of key enzymes in bacterial starch degradation systems are not unusual, and are frequently accompanied by increases in other proteins which form part of more complex machinery, such as in *Eubacterium rectale* [50]. Interestingly, pilin-related proteins (where vWA domain-containing proteins are found) were also amongst the most significantly upregulated proteins (Fig. 3a) (e.g. isopeptide-forming pilin-related protein, see Supplementary Table S8). These results suggest that these proteins play a key role in starch degradation and prompted further investigation into whether their transcripts are more highly expressed in the presence of RS.

### Clustered amylase genes are differentially expressed depending on starch structure

We investigated the regulation of the clustered amylase genes using transcriptomics in the presence of different starch structures. Total intracellular RNA of *B. globosum* 45 was measured in the presence of NMS, HylonVII®, glucose, or no substrate at 12 and 24 h. Principal component analysis showed that the largest source of observed variance in the transcriptome could be attributed to the presence of either NMS or HylonVII® (Fig. 3b). There was a minor distance separation observed between NMS and HylonVII® which prompted further direct comparisons.

Differential abundance analysis was performed to quantify gene transcript levels between conditions using the cut-off criteria of fold change ≥2 and false discovery rate (FDR) ≤0.1(Fig. 3c, Supplementary Fig. S3 and Supplementary Table S9). Notably, the three putative α-amylase genes ‘Ig-like domain containing proteins’ (alpha-amylase_110, alpha-amylase_162, alpha-amylase_720), were not significantly upregulated in the NMS condition compared to glucose at either time point. Though, a potential complementary protein, vWA protein_470, was significantly upregulated at 24 h in NMS. Highly abundant hydrolytic enzymes being constitutively expressed whilst complementary proteins being upregulated has been observed in other prominent starch-degrading bacteria [50].

In the presence of HylonVII® however, during mid-exponential growth (12 h), alpha-amylase_720 was more highly abundant compared to glucose but not significantly (fold-change = 1.38, FDR = 7.83E-05), along with another α-amylase at a different locus, WP_051592050.1, here called alpha-amylase_050 (fold-change = 1.20, FDR = 6.77E-13). At 24 h, however, all three α-amylase genes in the cluster, along with the vWA protein_470 (fold-change = 2.29, FDR = 3.81E-33) were significantly upregulated in line with the interesting growth curve observed (Fig. 2f). The clustered α-amylase proteins and vWA protein genes are not co-located in the genome.

The differential expression of these α-amylase genes at 24 h in HylonVII® suggests that starch structure induced a stronger transcriptional response. Comparing gene expression between HylonVII® and NMS, significant (<0.0013) differences were observed at 24 h (Fig. 3c). Additionally, significant differential expression of the α-amylase_720 and α-amylase_110 transcripts were observed in both conditions NMS and HylonVII® between the two time points (12 vs 24 h) (Fig. 3c). We report that α-amylase_720 is an interesting gene encoding a cell-wall-anchored, multi-modular α-amylase protein, accompanied by a CBM74 domain, and is co-transcribed with two other putatively starch-binding amylase-encoding genes. The gene cluster, appearing to be co-transcribed into proteins which could potentially form a system or complex on the cell surface, and would be a first for the genus of *Bifidobacterium*.

### Structural prediction and multiple sequence alignment of CBM74-containing amylase binding sites

**Figure 4:**
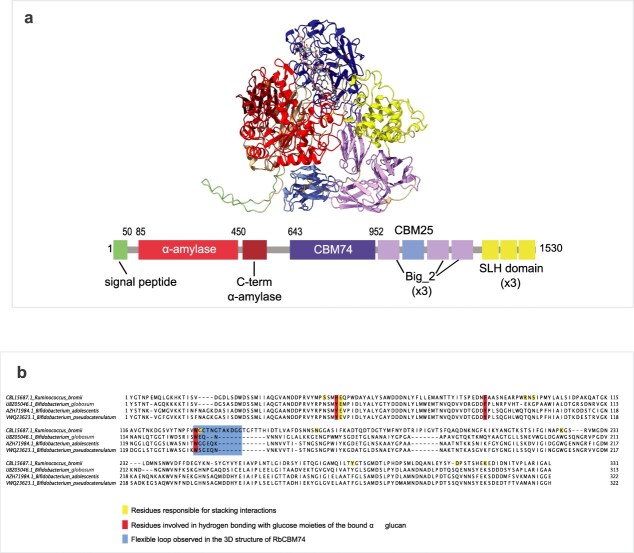
Structural prediction and multiple sequence alignment of CBM74-containing amylase binding sites. (a) The central protein (WP_099309720.1) is a large multi-modular protein; its 3D structure of is shown as predicted using AlphaFold2. It is predicted to contain α-amylase, CBM74, CBM25, and an S-layer homology domain. The gene cluster present in the *Bifidobacterium globosum* 45 genome containing 3 Ig-like fold containing proteins possess α-amylase (GH13) gene CAZyme predictions and occur at the same locus in the genome. Each α-amylase is also predicted to have at least one associated starch-binding carbohydrate module (CBM). (b) Multiple sequence alignment of representative bifidobacteria to the well-characterized CBM74 amylase protein in *Ruminococcus bromii* (CBL15687.1) originally performed by Photenhauer *et al.* Bacteria included (top to bottom) were *Bifidobacterium adolescentis* (AZH71984.1), *Bifidobacterium pseudocatenulatum* (VWQ23623.1), and *B. globosum* (UBZ05046.1). The key residues show are the stacking interactions in red and hydrogen bonding in yellow which enable binding between the amylase and starch; several key residues in the three bifidobacteria are largely conserved.

The primary protein in the discovered gene cluster is alpha-amylase_720. Alpha-amylase_720 possesses a signal peptide, suggesting extracellular export, and S-layer homology domains, which may anchor proteins to the bacterial G(+) cell wall (full predictions are detailed in Supplementary Table S10). Alpha-amylase_720 shares 81.5% amino acid identity and a very similar domain structure to the previously characterized resistant-starch amylase FMB-RSD3 from *B. choerinum,* and are therefore highly likely to be functional orthologs [32]. A possible 3D conformation of this enzyme was predicted using AlphaFold2 (Fig. 4a, Supplementary Fig. S4). Overlaying the recently solved structure of CBM74 from *Ruminococcus bromii* (termed RbCBM74) revealed the presence of a substrate analogue (a maltodecoase double helix) aligned in the CBM74 module-binding pocket, positioned near the active site of the α-amylase [46]. Through multiple sequence alignment of the RbCBM74 with *Bifidobacterium* CBM74 sequences we observed residue discrepancies between the three bifidobacterial enzymes and RbCBM74 in key enzyme-starch binding regions (Fig. 4b). The bifidobacterial enzymes shared residues in common for two of the three key protein regions whilst differing from RbCBM74 by one amino acid. One of these is involved in both stacking interactions between the enzyme and glucose molecules and hydrogen bonding of the bound α-glucan. The second is formative of the flexible loop region of the protein, which enables conformational changes and starch granule interaction; the bifidobacteria appear to be missing key residues from this region (Fig. 4b). The concordant amino acid residues within the bifidobacterial enzyme sequences are suggestive of a conserved binding and hydrolytic mechanism which differs from *R. bromii*.

### Infant isolate starch utilization depending on age and protein homology of discovered gene cluster

Orthologs of alpha-amylase_720 are almost exclusively found among members of the *Bifidobacterium* genus, including human isolates of *B. pseudocatenulatum* and *B. adolescentis*, each exhibiting protein sequence identities above 64.5% (Supplementary Table S11). The proteins in the gene cluster have statistically significant orthologs (51%–53% sequence identity) in the *B. pseudocatenulatum* LH662/659 strains from this study isolated from an exclusive breastfed infant at 5.2 months of life (Table 1). The activity of similar enzymes against RS has been confirmed previously in *B. adolescentis* 22L and *B. choerinum* FMB-1 [32]. Confirming that pre-weaning-age isolates lack genomic similarity to this gene cluster, there was low sequence coverage (8%–17%) for *B. longum* subsp. *longum* LH277 isolated from the same infant at 1.2 months of life (Table 1). Further, we report minor evidence of age-related bacterial starch utilization phenotypes (Supplementary Fig. S5) as we were interested in how bacterial phenotypes adapt to changing nutritional landscape in infancy [20, 29].

**Table 1:** Homology of gene cluster found *Bifidobacterium* strains isolated from the same infant at different time points BLAST® analysis of gene cluster sequence in *Bifidobacterium globosum* 45 (alpha-amylase_162, _720, and _110) against unique isolate whole genome sequences from a single, breastfed infant isolated at different age after birth.

**WP_051894162.1 (alpha_amylase_162)**					
	**Scientific name**	**Query cover**	**E value**	**% Identity**	
	*B. globosum* 45	100%	0	93.60	
	*Bifidobacterium choerinum* FMB-1	100%	0	79.80	
	*B. adolescentis* 22 L	82%	0	47.70	
	*Bifidobacterium pseudocatenulatum* YIT11027	66%	0	53.00	
	*Bifidobacterium pseudolongum* 44	100%	0	77.85	
Contig					Age (months)
LH277.19109_5_24.16	*Bifidobacterium longum subsp. longum* LH_277	17%	2.00E-07	50.90	1
LH277.19109_5_24.11	*B. longum subsp. longum* LH_277	12%	2.00E-07	26.73	1
LH659.19109_5_82.3	*B. pseudocatenulatum* LH_659	63%	0	53.00	5
ERS2658032	*B. pseudocatenulatum* LH_659	10%	7.00E-16	32.34	5
LH659.19109_5_82.4	*B. pseudocatenulatum* LH_659	39%	9.00E-11	28.64	5
LH659.19109_5_82.6	*B. pseudocatenulatum* LH_659	18%	1.00E-07	22.76	5
LH659.19109_5_82.3	*B. pseudocatenulatum* LH_662	63%	0	53.00	5
ERS2658032	*B. pseudocatenulatum* LH_662	10%	7.00E-16	32.34	5
LH659.19109_5_82.4	*B. pseudocatenulatum* LH_662	39%	9.00E-11	28.64	5
LH659.19109_5_82.6	*B. pseudocatenulatum* LH_662	18%	1.00E-07	22.76	5
**WP_099309720.1 (alpha_amylase_720)**					
	**Scientific name**	**Query cover**	**E value**	**% Identity**	
	*B. globosum* 45	100%	0	91.96	
	*B. choerinum* FMB-1	100%	0	75.61	
	*B. adolescentis* 22 L	87%	0	48.44	
	*B. pseudocatenulatum* YIT11027	73%	0	50.35	
	*B. pseudolongum* 44	77%	0	56.93	
Contig					Age (months)
LH277.19109_5_24.16	*B. longum subsp. longum* LH_277	8%	1.00E-07	50.88	1
LH659.19109_5_82.3	*B. pseudocatenulatum* LH_659	78%	0	50.35	5
LH659.19109_5_82.4	*B. pseudocatenulatum* LH_659	11%	7.00E-16	33.52	5
ERS2658032	*B. pseudocatenulatum* LH_659	12%	3.00E-12	26.70	5
LH662.19109_5_78.3	*B. pseudocatenulatum* LH_662	78%	0	50.35	5
LH662.19109_5_78.4	*B. pseudocatenulatum* LH_662	11%	7.00E-16	33.52	5
ERS2658033	*B. pseudocatenulatum* LH_662	12%	3.00E-12	26.70	5
**WP_162288110.1 (alpha_amylase_110)**					
	**Scientific name**	**Query cover**	**E value**	**% Identity**	
	*B. globosum* 45	100%	0	88.57	
	*B. choerinum* FMB-1	100%	0	69.00	
	*B. adolescentis* 22 L	84%	0	42.73	
	*B. pseudocatenulatum* YIT11027	60%	6.00E-161	52.40	
	*B. pseudolongum* 44	95%	6.00E-142	49.23	
Contig					Age (months)
LH277.19109_5_24.16	*B. longum subsp. longum* LH_277	12%	2.00E-08	48.57	1
ERS2658037	*B. longum subsp. longum* LH_277	9%	0.001	27.48	1
LH659.19109_5_82.3	*B. pseudocatenulatum* LH_659	67%	6.00E-163	52.21	5
LH659.19109_5_82.4	*B. pseudocatenulatum* LH_659	18%	6.00E-11	30.46	5
ERS2658032	*B. pseudocatenulatum* LH_659	10%	3.00E-07	25.33	5
LH662.19109_5_78.3	*B. pseudocatenulatum* LH_662	67%	6.00E-163	52.21	5
LH662.19109_5_78.4	*B. pseudocatenulatum* LH_662	18%	6.00E-11	30.46	5
ERS2658033	*B. pseudocatenulatum* LH_662	10%	3.00E-07	25.33	5

The BLAST® results from *Bifidobacterium adolescentis* 22L are displayed for comparison.

## Discussion

Starch structure significantly influences the growth and metabolite output of saccharolytic bacteria, which impacts microbiome ecology [51, 52]. The *Bifidobacterium* strains tested displayed slower fermentation dynamics and prolonged acetate production when grown in HylonVII®, a high-amylose RS. This could have important implications for the microbiome, as prolonged acetate release may enhance competitive exclusion of pathogenic bacteria and support beneficial microbial populations [11]. We investigated two closely related but phenotypically distinct isolates *B. pseudolongum* 44 and *B. globosum* 45 to uncover a new gene cluster pertinent to the RS degradation. Both isolates were predicted to encode several amylase genes, including extracellular starch-binding amylases as well as intracellular enzymes that target glycosidic bonds after oligosaccharides are imported into the cell. One gene had a predicted CBM74 module common in multi-domain α-amylases. CBM74 modules can synergize with flanking CMB25 or CBM26 domains enabling effective granule docking [16, 46, 53]. In *R. bromii*, CBM74 recognizes longer single- and double-helical α-glucans whilst flanking CBM26 binds shorter malto-oligosaccharides [46]. Of the 71 gut microbes that encode CBM74 family modules, 50 belong to the genus *Bifidobacterium* [16]. Relatively few investigations have explored bifidobacteria that contain CBM74 domain-containing amylases and their ability to enhance RS binding or degradation [32]. Hybrid metagenome assemblies have confirmed a higher abundance of CBM74 sequences in response to RS being present in an *in vitro* fermentation model [52]. In our study, dbCAN2 annotations revealed three ‘Ig-like domain-containing protein’ genes in the stronger RS-degrading isolate *B. globosum* 45. These proteins share homology with other previously described enzymes in other species [32] and not in a clustered arrangement as we have shown here. Ig-like domain proteins have been described as important for substrate binding in other gut members such as *Roseburia inulinovorans* and *Eubacterium rectale* [16]. These bacteria produce a cell-wall anchored GH13 amylase with at least one CBM26 domain and Ig-like domains, which may function as unidentified starch-specific CBMs or play a structural role in degradation. In the prolific carbohydrate degrader *Bacteroides thetaiotaomicron*, elements of its large starch uptake system contain several starch-binding domains with a canonical Ig-like/β-sandwich fold attached to amylases [16].

These clustered, Ig-like fold domain-containing proteins were expressed in both starch and glucose conditions. We observed a growth pattern with two exponential growth phases which may occur when the presence of RS triggers the transcription and translation of amylolytic molecular machinery. Furthermore, *B. globosum* 45’s metabolism of HylonVII® resulted in a higher degree of transcription of the clustered α-amylases compared to normal maize starch, indicating that their expression could be influenced by starch structure, potentially through prolonged exposure to imported starch degradation products, though, we have not investigated the mechanisms of transcriptional regulation. Our proteomics results showed a modest, statistically significant upregulation of amylases in normal maize, while gene transcription was not significantly affected by the same starch. Modest upregulation could be due to multiple factors: (i) constitutive gene expression, (ii) in the presence of starch the half-life of the amylases increases (possibly enhanced by CBM74 binding to double-helical starch), (iii) starch binding enhances transcription and translation of a secondary proteins binding to and stabilizing amylases [54]. Indeed, we observed co-upregulation of some secondary proteins—pilins, vWA proteins—which play complementary roles in starch hydrolysis in *E. rectale* [50]. These proteins can act as binding proteins when upregulated in the presence of amylase and starch [54], and may also facilitate the assembly of enzyme complexes [55–57]. Further research is needed to elucidate the specific functions of vWA proteins and pilins in bifidobacterial starch metabolism.

We report that the clustered amylase genes discovered in ruminant isolate *B. globosum* 45 are potentially important because we identified orthologs of this gene cluster in infant *B. pseudocatenulatum* strains. The effect of dietary components, age, and strain characteristics within the gut microbiota is highly relevant during infancy, a critical period of microbiome maturation [21, 23, 58]. As the infant digestive system develops, its ability to digest dietary components becomes more efficient but is influenced by individual variation [59]. The introduction of solid foods during weaning, especially starchy substrates, stimulates the expansion of certain key taxa that promote microbiota development [23]. Previous research has shown that *Bifidobacterium* strains in breastfed infants engage in cross-feeding interactions, where HMO-degrading strains support the growth of non-degrading strains [3]. In this work, using the same culture collection of *B. breve* and *B. pseudocatenulatum* strains we showed that weaning-age (5+ months) *Bifidobacterium* strains also possessed the capacity to degrade starch substrates. They were able to generate maltose and acetate as metabolic by-products, while strains that had been isolated before weaning-age could not. Whilst *B. pseudocatenulatum*’s RS degradation was not as efficient as other species tested (*B. breve*), we propose that prolonged degradation and lower import of maltose may benefit the ecosystem. These strains had the highest number of CAZymes from a variety of families such as the starch-related GH13, CBM48, CBM25, as well as GH43 which includes enzymes acting on a broad spectrum of substrates, providing further evidence of bacterial generalism [60]. Possessing a large variety of enzymes would make them well suited for weaning, which is interesting given that the isolates were from pre-weaning, fully-breastfed infants. The presence of α-amylases appears to be specific to the strain(s) used in this study, as they are not typically found in this species. *Bifidobacterium pseudocatenulatum* as a species has also been demonstrated to function as metabolic generalists which facilitates microbiome stability [61]. These findings highlight a potential bridging role for *Bifidobacterium pseudocatenulatum* during the transition from milk to solids.

The clustered amylase genes identified have co-regulated expression, are more highly transcribed in the presence of RS expression, and are linked to upregulation of pilin proteins highlighting a potentially new mechanism of starch metabolism. This study provides new insights into how *Bifidobacterium* interact with dietary starch. The early-life microbiota may possess bifidobacteria with multi-tasking metabolic capabilities, including starch—a key weaning food—metabolism. The discovery of a novel amylase gene cluster and the importance of CBM74 in starch binding has the potential to extend our understanding of *Bifidobacterium*’s role in shaping the gut microbiome during weaning. These findings have implications for the development of bifidogenic functional foods, including the incorporation of RS, synbiotics, and strategies for promoting gut health throughout life.

There are limitations in this study that should be considered. In favour of in-depth research into molecular mechanisms, we focused on a small set of human and animal bacterial species and strains; hypotheses proposed here could be tested using more pre- and post-weaning isolate pairs from the same infant. The health benefits of RS degradation and acetate production by these strains were not explored *in vivo* or using cell culture methods as this was considered to be outside the scope of this work. Finally, the role of vWA protein was not resolved and could be the subject of future research.

## Supplementary Material

Supplementary_Table_S1_Strain_information_ycag136

Supplementary_Table_S2_LH_strains_metadata_ycag136

Supplementary_Table_S3_Figure_1_dbCAN2_Results_ycag136

Supplementary_Table_S4_Figure_2_Statistics_ycag136

Supplementary_Table_S5_dbCAN_Tesults_Bp44_and_Bg45_ycag136

Supplementary_Table_S7_Bg45_Gene_cluster_ycag136

Supplementary_Table_S8_Proteomics_data_ycag136

Supplementary_Table_S9_RNA_Full_Volcano_Plots_ycag136

Supplementary_Table_S10_Bg45_gene_ycag136

Supplementary_Table_S11_Homology_of_Ig_protein_v2_ycag136

Supplementary_Figures_S1-S5_final_May2026_ycag136

renamed_c7e7e_ycag136

## Data Availability

The RNA-seq raw read data can be found in the National Centre for Biotechnology Information (NCBI) database under BioProject ID: PRJNA1156008. The mass spectrometric raw files and the MaxQuant output files have been deposited to the ProteomeXchange Consortium via the PRIDE partner repository [62] and can be accessed using the identifier PXD056548 (https://proteomecentral.proteomexchange.org/cgi/GetDataset?ID=PXD056548). The genomic data underlying this article are available in the NCBI repository. A full list of each bacterial strain and its accession number can be found in Supplementary Table S1. The remaining data underlying this article are available in the article and within its online supplementary material.

## Subject ontology

Bifidobacteria; Bifidobacterium; resistant starch; alpha amylase; CAZyme; microbiome; microbiota; weaning
