# Supplementary material for: Bifidobacterial genes upregulated by resistant starch investigated using multi-omics have orthologs in infant gut isolates
Source: ISME Commun. 2026 May 17;6(1):ycag136. doi: 10.1093/ismeco/ycag136 (PMC13271388; doi:10.1093/ismeco/ycag136)
Supplement: Supplementary_Figures_S1-S5_final_May2026_ycag136 [file supplementary_figures_s1-s5_final_may2026_ycag136.pdf]

Supplementary Figure S1

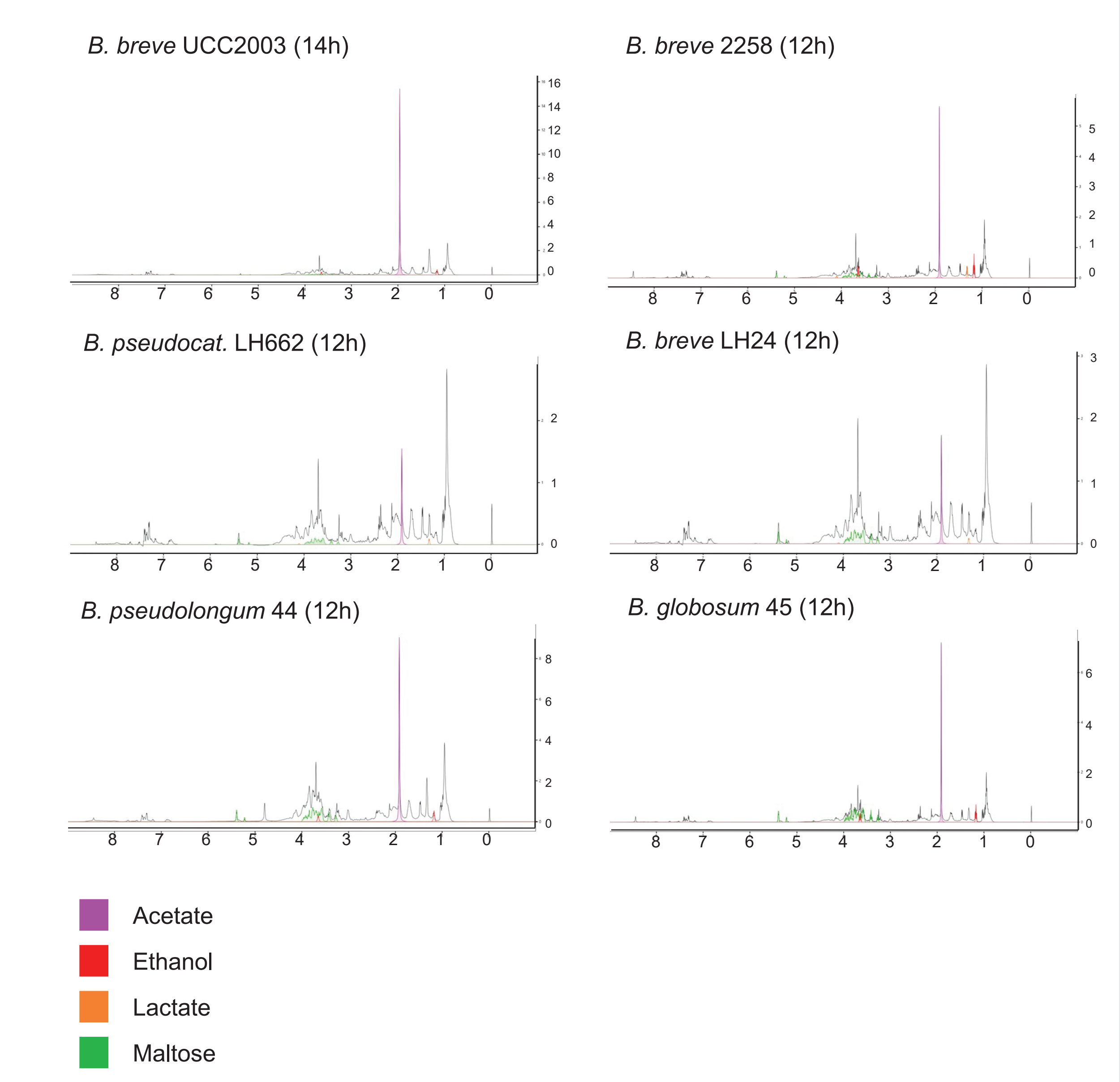

Supplementary Figure S1 NMR spectra of bacterial isolates grown in normal maize starch

Representative NMR spectra from one replicate of each strain at 12-14h culture time where the bacterial were all grown in mMRS + 1% normal maize starch (NMS). The key metabolites shown were selected due to their consistent and reproducible increases in concentration in response to starch across replicates and are highlighted with colour are: acetate (purple), ethanol (red), lactate (orange), maltose (green). The most relevant to hypotheses surrounding starch structure and bacterial enzymatic hydrolysis was maltose, whilst the most relevant and consistent indicator of bacterial metabolism of the substrate was acetate.

Supplementary Figure S2

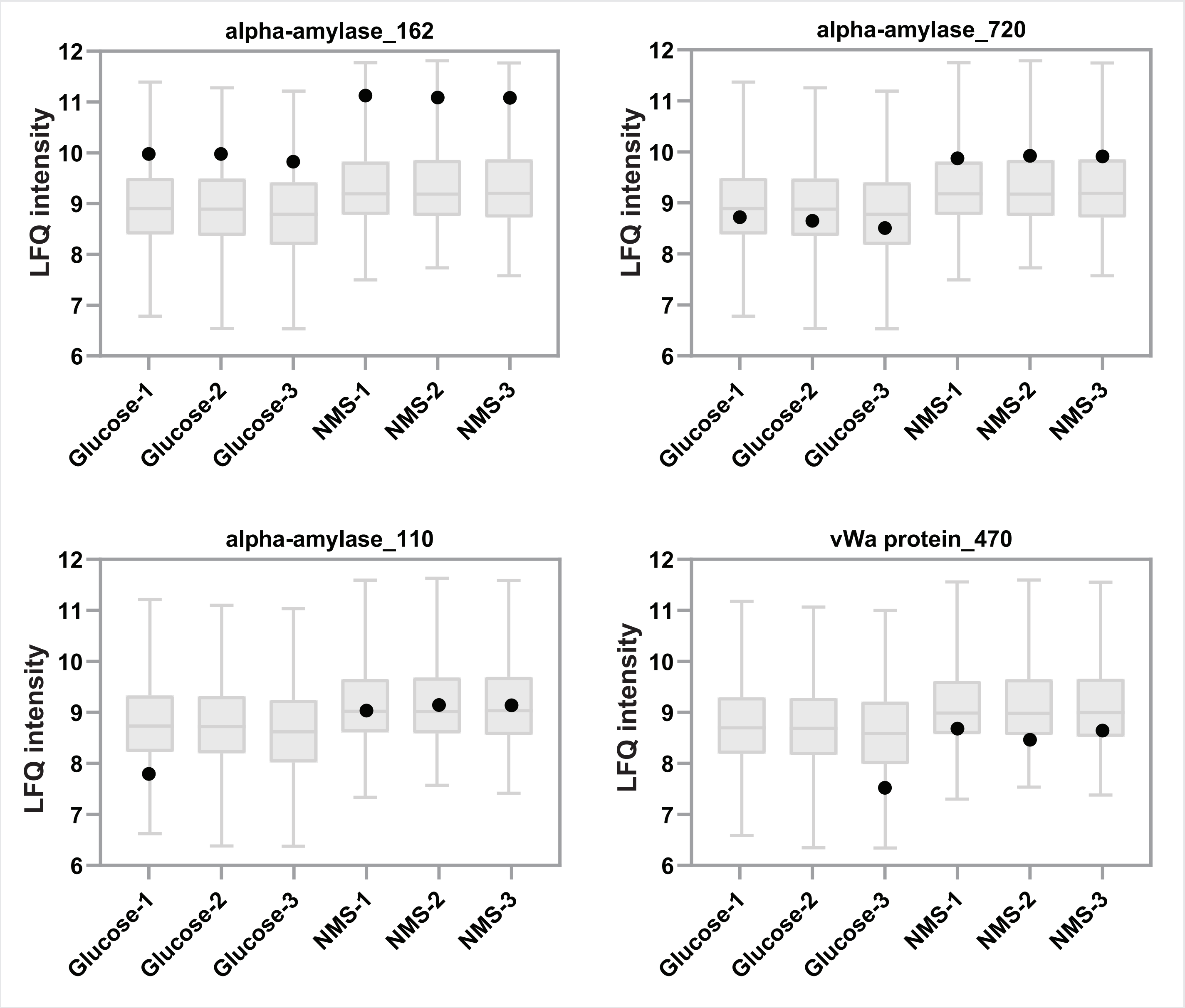

Supplementary Figure S2 Raw LFQ intensities of individual proteins of interest

The Label-Free Quantification (LFQ) intensities of each alpha-amylase in the novel gene cluster (alpha-amylase_162, _720, and _110) and a vWa protein_470 found to be upregulated in the normal maize starch (NMS) condition compared to glucose being present in mMRS medium. Each culture condition was performed in triplicate: each replicate is displayed as a single point on each plot, with the boxplot in the background being the overall protein expression in each condition, with median and quantiles displayed.

Supplementary Figure S3

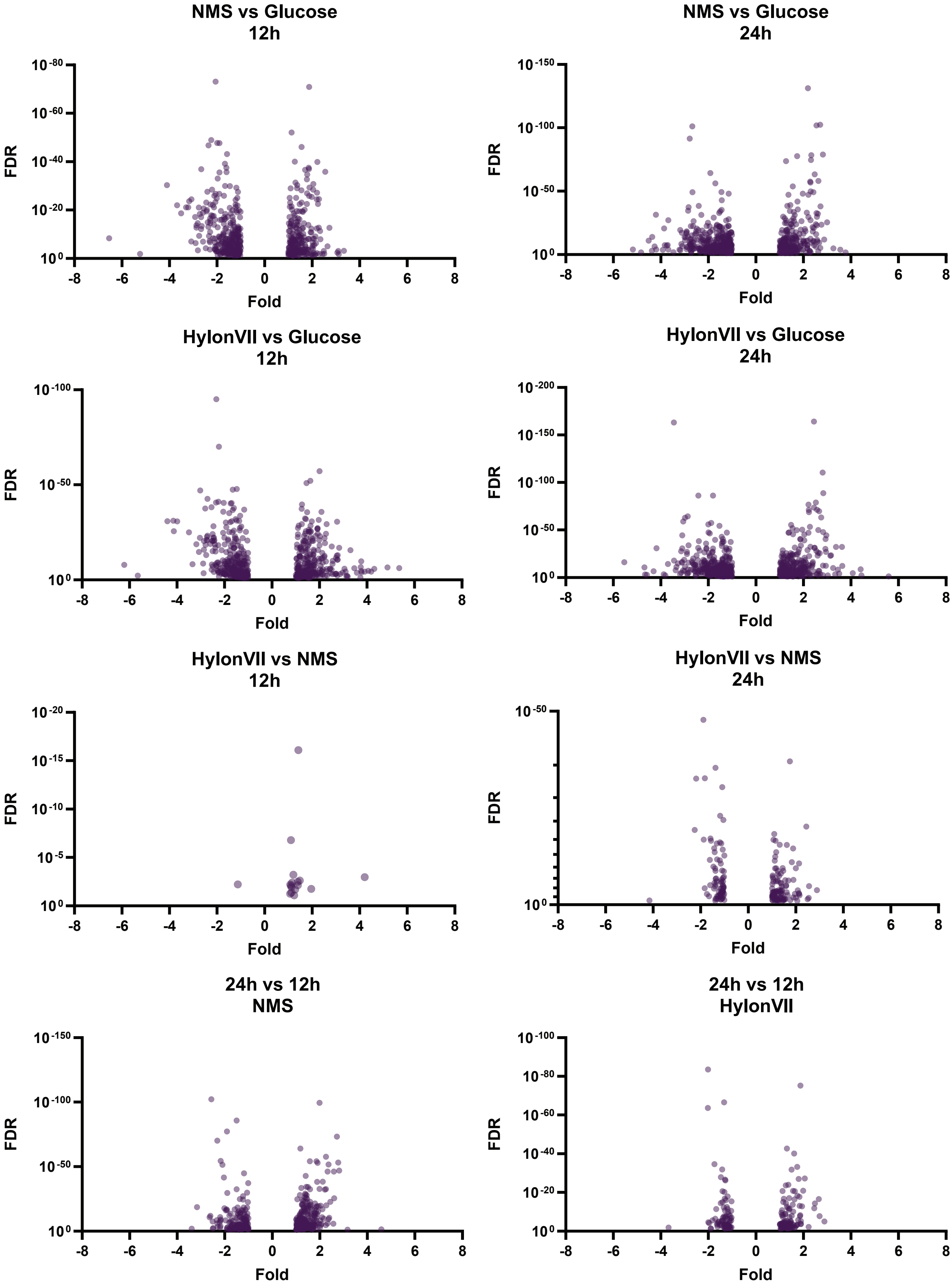

Supplementary Figure S3 Transcriptome volcano plots of all up- and down-regulated genes from differential gene abundance analysis

DESeq2 was used to analyse differential gene expression under two conditions e.g. NMS vs Glucose at equivalent w/v % supplemented in mMRS media. The down-regulated genes (left side of the plot) and up-regulated genes (right side of the plot) are displayed alongside their false discovery rate (FDR). The first condition in the plot title e.g. NMS vs Glucose, NMS is the primary condition (left side of the plot = down-regulated genes in the presence of NMS compared to Glucose; right, up-regulated in the presence of NMS compared to Glucose). A full list of genes, their fold-change, and FDR can be found in Supplementary Table S9.

Supplementary Figure S4

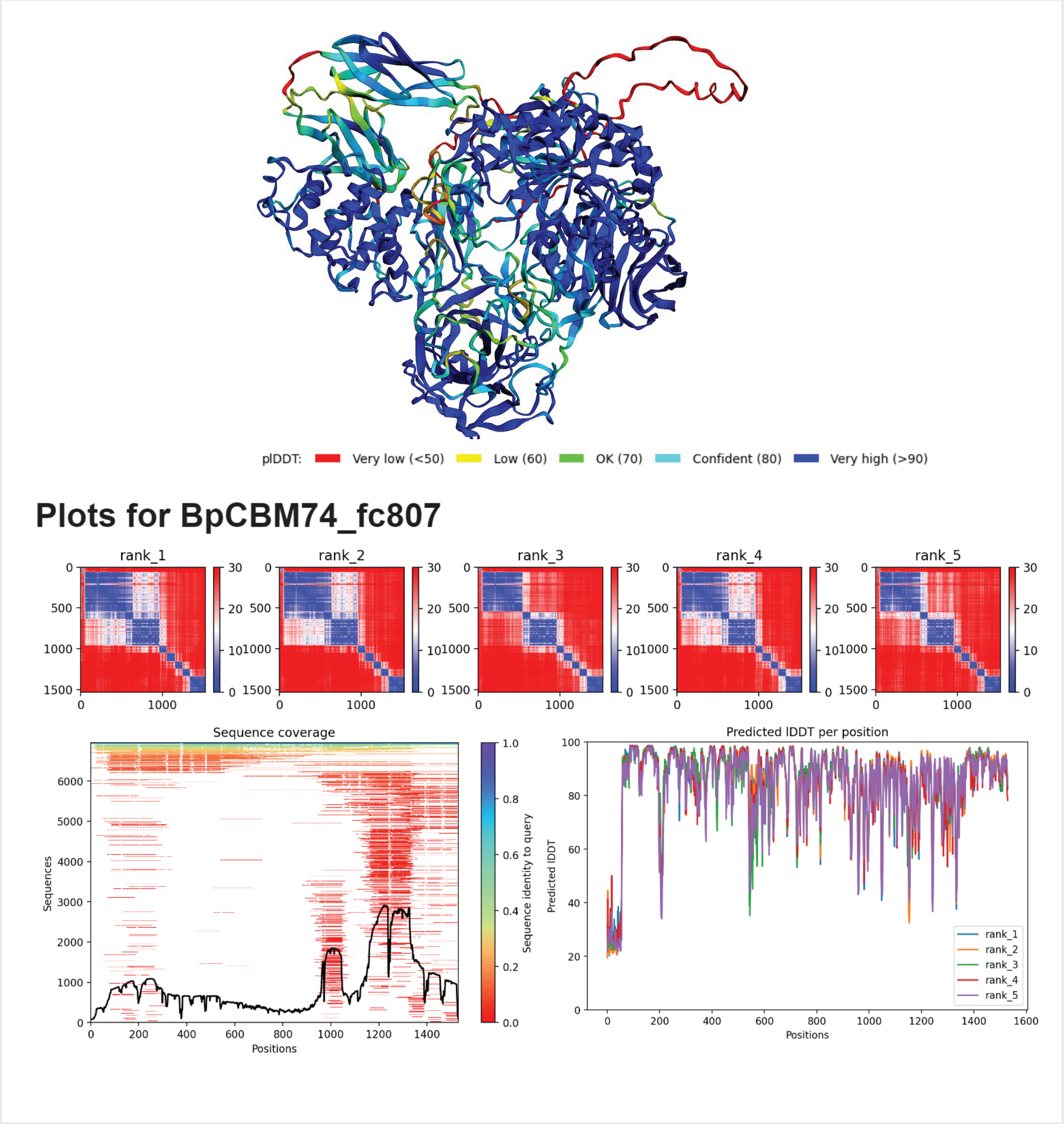

Supplementary Figure S4 AlphaFold2 confidence prediction metrics

Confidence metrics provided for AlphaFold2 sequence mapping of *B. globosum* alpha-amylase_720 CBM74 domain was structurally aligned with the X-ray crystal structure of CBM74 from *Ruminococcus bromii.*

Supplementary Figure S5

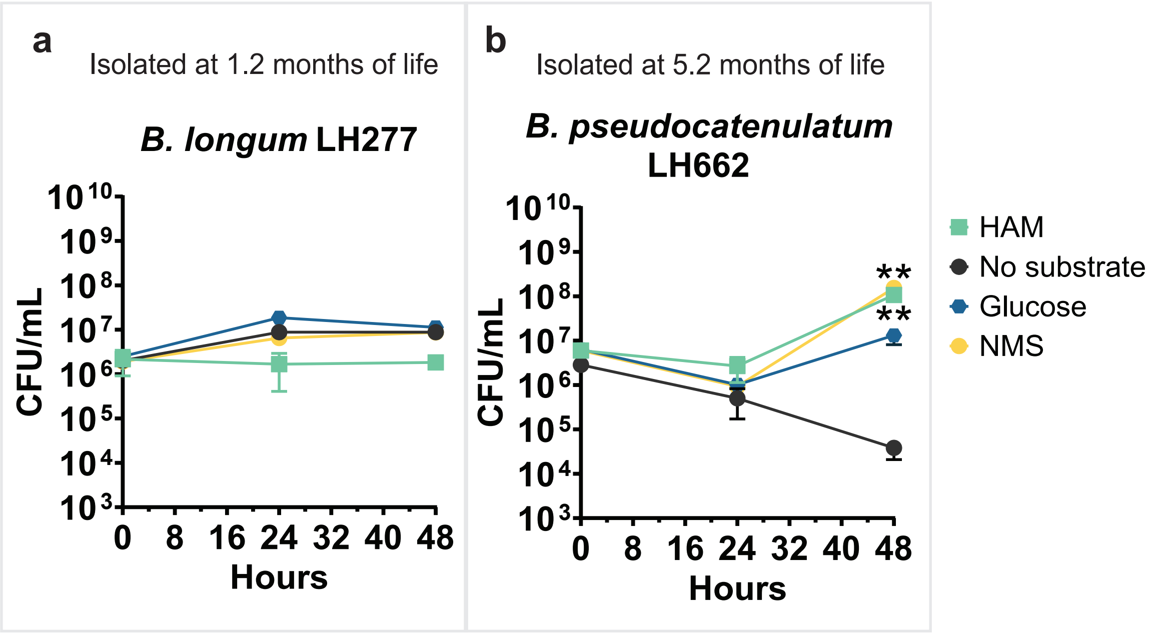

Supplementary Figure S5 Phenotypic analysis of two *Bifidobacterium* isolates from the same infant at different time points

Growth of *Bifidobacterium* isolates in the presence of 1% w/v normal maize starch (NMS), high (50%) amylose maize (HAM), 1% w/v D-glucose, and no substrate in modified MRS media. The isolates tested were obtained at different ages of the same infant: *B. longum* subsp. *longum* LH277 at 37 days after birth; *B. pseudocatenulatum* 662 at day 159. All data were generated in triplicate; error bars denote SD. Dunnett's multiple comparisons tests were performed to compare the CFU/ml mean values of the starch conditions to the no substrate (negative) control mean values. Significance values: * <0.05, ** <0.01, ** < 0.001, and **** <0.0001.

a) *B. longum* subsp*. longum* LH277 had significantly lower CFU/ml in the presence of high amylose maize (HAM) starch and no significant growth in the presence of NMS compared to the negative control with no added carbon source.

b) *B. pseudocatenulatum* LH662 was capable of degrading both NMS and HAM.
